# Supplementary material for: XANES Measurements for Studies of Adsorbed Protein Layers at Liquid Interfaces
Source: Materials (Basel). 2020 Oct 17;13(20):4635. doi: 10.3390/ma13204635 (PMC7603035; doi:10.3390/ma13204635)
Supplement: Supplementary file 1 [file materials-13-04635-s001.docx]

Supplementsry Materials

XANES Measurements for Studies of Adsorbed Protein Layers at Liquid Interfaces


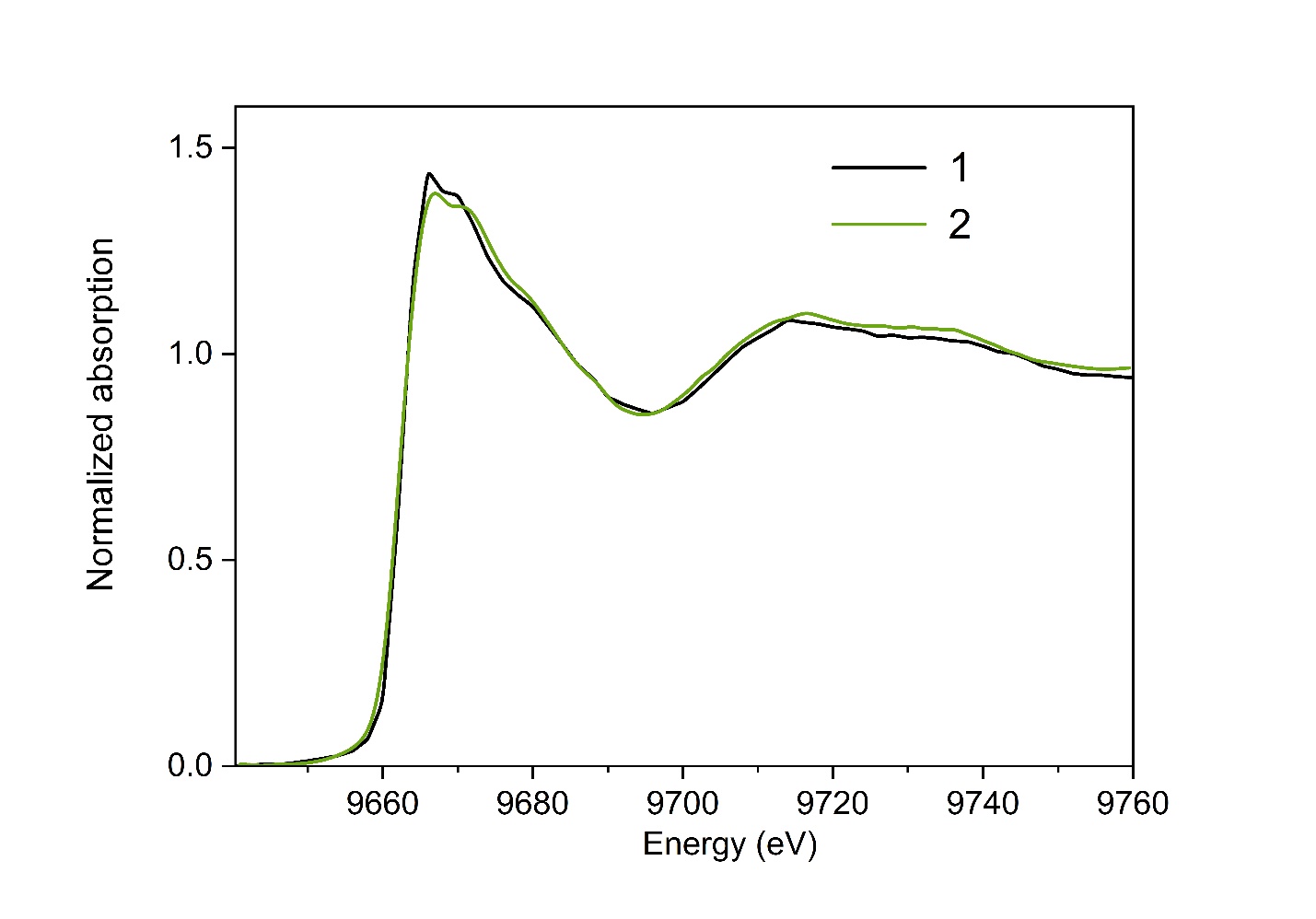


**Figure S1.** Comparison of XANES experimental data for the parkin protein layer obtained at the ID10 beamline, ESRF (curve 1) under “mild” conditions and those recorded at the at the LANGMUIR beamline, Kurchatov center for synchrotron radiation (curve 2).

**Publisher's Note:** MDPI stays neutral with regard to jurisdictional claims in published maps and institutional affiliations.

| 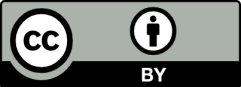 | © 2020 by the authors. Submitted for possible open access publication under the terms and conditions of the Creative Commons Attribution (CC BY) license (http://creativecommons.org/licenses/by/4.0/). |
| --- | --- |
